# Cardioprotective effect of ritonavir, an antiviral drug, in isoproterenol induced myocardial necrosis: a new therapeutic implication

**DOI:** 10.1186/1479-5876-11-80

**Published:** 2013-03-26

**Authors:** Prachi Gupta, Abhinav Kanwal, Uday Kumar Putcha, Yogesh Bulani, Bhavesh Sojitra, Tarak Nath Khatua, Madhusudana Kuncha, Sanjay Kumar Banerjee

**Affiliations:** 1 Division of Medicinal Chemistry and Pharmacology, Indian Institute of Chemical Technology (IICT), Hyderabad, India; 2 Department of Pathology, National Institute of Nutrition, Hyderabad, India

**Keywords:** Isoproterenol, Necrosis, SGLT1, Oxidative stress, GLUT, Ritonavir, Phlorizin

## Abstract

**Background:**

Ritonavir is a HIV protease inhibitor. In addition to its antiviral effect, Ritonavir directly inhibits the insulin-regulated glucose transporter GLUT4 and blocks glucose entry into fat and muscle cells. However, the effect of Ritonavir on cardiac GLUT4 inhibition during myocardial necrosis is not investigated. In the present study, we evaluated the role of Ritonavir in isoproterenol-induced myocardial necrosis *in vivo* and compared the effect with Phlorizin, a nonslective SGLTs inhibitor.

**Methods:**

Isoproterenol (ISO) (150 mg/kg/day, i.p for 2 consecutive days) was administered to mice to cause myocardial necrosis. Phlorizin (400 mg/kg/day i.p twice daily for 2 days) and Ritonavir (10 mg/kg/day i.p twice daily for 2 days) were administered in two different groups of mice before isoproterenol administration.

**Results and discussion:**

Isoproterenol (ISO) (150 mg/kg/day, i.p for 2 consecutive days) administration caused significant (p < 0.05) increase in heart/body weight ratio, and myocardial necrosis as evident by significant (p < 0.05) increase in serum markers i.e. SGOT and CK; and cardiac histopathological changes. Significant (p < 0.05) reduction in myocardial SOD and catalase activities, and GSH level along with a significant (p < 0.05) rise in myocardial TBARS and nitric oxide levels were observed after ISO administration. However, administration of phlorizin, a SGLT1 inhibitor has been found to exhibit partial protection in ISO induced myocardial necrosis, as observed by significant decrease in heart/body weight ratio and myocardial nitric oxide level; significant increase in myocardial SOD and catalase activities along with no histopathological alterations. On the other hand, administration of ritonavir, a nonspecific GLUT inhibitor has been found to exhibit complete protection as observed by normalisation of heart/body weight ratio, serum markers, antioxidant enzymes activities and histopathological alterations. *In vitro* study with heart homogenate confirmed no antioxidant effect of ritonavir and phlorizin in the absence and presence of isoproterenol.

**Conclusions:**

Our study concluded that ritonavir, a nonspecific GLUT inhibitors showed complete protection in catecholamine induced myocardial necrosis.

## Introduction

Myocardial infarction (MI) is one of the main causes of death from cardiovascular disease. MI is defined as an acute condition of necrosis of the myocardium that occurs as a result of imbalance between coronary blood supply and myocardial demand [1]. The heart is capable of utilizing a variety of metabolic substrates and is able to switch rapidly depending on pathological and physiological conditions. Although, glucose and fatty acid are mainly used as fuel for energy in the heart, fatty acid remains a major source of energy [2]. However, in an ischemic heart due to less availability of oxygen, glucose becomes the major source of energy, as glycolysis switches from aerobic to anaerobic conditions. Such metabolic perturbation is seen in various cardiac diseases which result in shifting of metabolic utilization of substrates toward glucose from fatty acids [3].

In mammalian heart, glucose transport is believed to be mediated mainly by two members of the GLUT family, GLUT1 and GLUT4. Increased activity of GLUTs in diseased heart has been reported earlier [2]. SGLT is another group of glucose transporter which is mainly involved in absorption of glucose from renal and intestinal tissue. It was reported that SGLT1 is 10 times more expressed in human cardiomyocytes than kidney tissue [4] and their increased cardiac expression in different heart diseases in mice as well as in human has been reported recently [5,6]. However the role of SGLT1 in heart is still not clear. Thus further investigating the role of GLUTs and SGLT1 inhibitors during cardiac injury and their comparison is of great importance.

In the present study, we inhibited SGLTs and GLUTs transporters in heart by using pharmacological inhibitors, phlorizin (nonselective SGLT blocker) and ritonavir (nonselective GLUTs blocker) respectively, and evaluated the relative importance of these transporters during myocardial necrosis induced by isoproterenol.

## Materials and methods

### Animals

Male Swiss albino mice (20-25 gm) were provided by National Institute of Nutrition (NIN), Hyderabad. The animals were housed in BIOSAFE, an animal quarantine facility of Indian Institute of Chemical Technology (IICT) Hyderabad. The animal house was maintained at temperature 22 ± 2°C, relative humidity 50 ± 15% and 12 hour dark/light cycle was maintained throughout the study. Mice had free access to food (pellet diet supplied from NIN, Hyderabad) and water *ad libitum.* All animal experiments were undertaken with the approval of Institutional Animal Ethics Committee of Indian Institute of Chemical Technology, Hyderabad, India.

### Experimental protocols

Weight matched male swiss albino mice were randomly divided into four groups with each group having eight animals. Six and two animals from each group were kept for biochemical and histopathological evaluation, respectively. The doses used in this study were selected on the basis of reports of previous studies [7,8].

•Control group (IP injection of physiological saline and vehicle 0.2 ml/day).

•ISO group (SC injection of ISO 150 mg/kg/day for 2 consecutive days).

•ISO+Phz group (IP injection of phlorizin 400 mg/kg/day 10 min. prior to ISO dose for 2 days).

•ISO+RTV group (IP injection of ritonavir 10 mg/kg/day 10 min. prior to ISO dose for 2 days).

ISO is dissolved in PBS while phlorizin and ritonavir were dissolved in vehicle (75% PBS +15% DMSO + 10% absolute alcohol). Control group received phosphate buffer saline (PBS) and vehicle at the time of ISO and ‘phlorizin and ritonavir’ administration, respectively. ISO group received vehicle at the time of phlorizin and ritonavir administration.

### Sample collection and biochemical assay

The animals in all groups were sacrificed 48 hrs after first dose of isoproterenol injection. Cardiac tissues were collected and stored at - 80°C for further biochemical evaluation. At the time of sacrifice, blood was collected by cardiac puncture, serum was separated by centrifugation at 4000 rpm (4°C) for 15 minutes and serum markers (SGOT and CK) were analysed by auto blood analyser (Bayer diagnostic). SGOT and CK were expressed in IU/L.

### Assessment of biochemical parameters

Each heart was homogenized with 20 times volume of heart weight in ice cold 0.05 M potassium phosphate buffer and treated separately as described below for the measurement of different biochemical parameters [9]. 20% homogenate was diluted with 10% trichloro acetic acid (TCA) in 1:1 ratio then centrifuged at 5000 rpm for 10 min. Supernatant was separated for GSH estimation as described [10]. Rest 80% homogenate was centrifuged at 15,000 rpm for 60 min. Supernatant was separated for estimation of nitric oxide (Nitric oxide assay kit, Assay Design), superoxide dismutase (SOD) (SOD kit, Fluka) and catalase [11]. Pallets from both homogenates were taken and resuspended in 1 ml of 10% TCA solution for TBARS estimation as earlier described [12].

### Histopathological studies

All cardiac samples after euthenisation were fixed in 10% neutral buffer formalin. Paraffin embedded 5 μm thick sections were obtained and stained with Hematoxylin and Eosin (H&E stain). Prepared sections were examined under light microscope to assess gross myocyte injury and the effects of interventions.

### In vitro antioxidant assay

Adult male swiss albino mice were euthanized. Heart was excised, washed with 0.9% NaCl solution and homogenised with 20-times volume of heart weight in 0.05 M ice-cold phosphate buffer [pH 7.4] [13]. Heart homogenate (0.25 ml) was mixed with 0.1 ml of 0.05 M phosphate buffer (pH 7.4), 0.05 ml of 0.1 mM ascorbic acid, 0.05 ml of 4 mM FeCl2 solution and 0.05 ml of the test sample. The mixture was incubated at 37°C for 1 hour and estimated for thiobarbituric acid reactive substances (TBARS). TBARS levels in heart homogenate were measured after treatment with phlorizin (450 μM) and ritonavir (15 μM) in presence and absence of isoproterenol (1.0 M). Data were expressed as nanomoles/ml homogenate using extinction co-efficient of MDA (1.56 × 10^-5^ M^-1^ cm^-1^).

### Statistical analysis

All values were expressed as mean ± SEM. Data were statistically analyzed using one way ANOVA for multiple group comparison, followed by student unpaired ‘t’ test for group wise comparison. Significance was set at P ≤ 0.05. Data were computed for statistical analysis by using Graph Pad Prism Software.

## Results

### Heart weight / Body weight Ratio

A significant (p ≤ 0.001) increase in heart weight / body weight ratio was observed in ISO group in comparison to control. However, significant (p ≤ 0.05) decrease in heart weight / body weight ratio was observed both in ISO + Phz and ISO + RTV groups in comparison to ISO group (Table 1).

**Table 1:** Heart weight / body weight ratio in different experimental groups

**Groups**	**Control**	**ISO**	**ISO + Phz**	**ISO + RTV**
**Heart weight / Body weight × 10**^**-3**^	5.33 ± 0.11	6.38 ± 0.20^***^	5.89 ± 0.13^†^	5.57 ± 0.09^†††^

All the values were expressed Mean ± SEM, (N = 8) ***p < 0.001 vs. control group; ^†^p < 0.05, ^†††^p < 0.001 vs. ISO group.

### Serum parameters

SGOT and CK levels were significantly increased (*p* < 0.05) in ISO group in comparison to control group. Significant (*p* < 0.05) increase in SGOT level but no change in CK level was observed in ISO + Phz group. However, significant (*p* < 0.05) decrease in both SGOT and CK levels was observed in ISO + RTV group in comparison to ISO group (Table 2).

**Table 2:** Serum SGOT and CK activities in different experimental groups

**Group**	**SGOT (IU/L)**	**CK (IU/L)**
Control	62.12 ± 5.03	287.66 ± 33.08
ISO	79.20 ± 6.52*	474.00 ± 63.28**
ISO + Phz	104.66 ± 4.78^††^	495.01 ± 130.57
ISO + RTV	65.80 ± 4.33^†^	301.80 +5.51^†^

All the values were expressed Mean ± SEM, (N = 6) *p < 0.05, **p <0.01, vs Control group; ^†^p < 0.05, ^††^p < 0.01, vs ISO group.

### Myocardial TBARS and Nitric oxide

Myocardial TBARS and nitric oxide levels were significantly increased (*p* < 0.01) in ISO group in comparison to control. There was significant (*p* < 0.05) decrease in myocardial nitric oxide level but no change in myocardial TBARS level in ISO + Phz group in comparison to ISO group. However, there was significant (*p* < 0.05) decrease in both myocardial TBARS and nitric oxide level in ISO + RTV group in comparison to ISO (Table 3).

**Table 3:** Myocardial TBARS, Nitric oxide, GSH, Catalase and SOD in different experimental groups

**Groups**	**TBARS (nmoles / gm heart wt.)**	**Nitric oxide (μmole / mg protein)**	**GSH (mg / gm heart wt.)**	**Catalase (units / mg protein)**	**SOD (units / mg protein)**
Control	27.99 ± 4.25	0.018 ± 0.001	4.22 ± 0.45	7.82 ± 0.15	313.89 ± 13.69
ISO	47.81 ± 5.70**	0.023 ± 0.001**	3.05 ± 0.09*	5.60 ± 0.14***	245.33 ± 7.45**
ISO + Phz	36.40 ± 6.58	0.019 ± 0.001^†^	3.37 ± 0.59	7.98 ± 0.52^††^	320.42 ± 12.44^††^
ISO + RTV	28.93 ± 4.03^†^	0.018 ± 0.001^†^	4.75 ± 0.72^†^	7.95 ± 0.49^††^	372.41 ± 18.80^†††^

All the values were expressed Mean ± SEM, (N = 6) *p < 0.05, **p <0.01, ***p <0.001 vs Control group; ^†^ p < 0.05, ^† †^ p < 0.01, ^† † †^ p < 0.001 vs ISO group.

### Myocardial GSH, catalase and superoxide dismutase (SOD)

Myocardial GSH, catalase and SOD were significantly decreased (*p* < 0.05) in ISO group in comparison to control group. There was significant (*p* < 0.01) increase in both myocardial catalase and SOD level but no significant change in myocardial GSH level in ISO + Phz group in comparison to ISO. However, significant (*p* < 0.05) increase in all three parameters was observed in ISO + RTV group in comparison to ISO (Table 3).

### Histopathology

**Figure 1:**
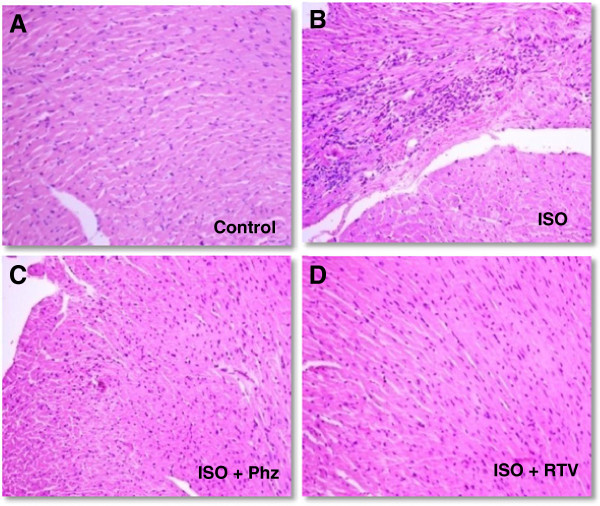
**Histopathological changes in mice heart (n = 2). A**. Control group: myocardial structure is intact with cardiomyocytes lined up in order (HE, 200X). **B** ISO group: photomicrograph showing formation of intracytoplasmic vacuolization, myocytolysis with diffused lymphocytes and monocytes infiltration (HE, 200X). **C**. ISO + Phz group: Photomicrograph of cardiac muscle showing normal architecture (HE, 200X). **D**. ISO + RTV group: Photomicrograph of cardiac muscle showing normal architecture (HE, 200X).

H&E sections from ISO group showed the features of severe inflammation along with presence of inflammatory cells, vacuolization of myocytes and focal areas of myocytolysis. However, H&E sections from ISO + phz and ISO + RTV groups showed no signs of any injury (Figure 1).

### In vitro antioxidant effect

*In vitro* antioxidant assay was performed with heart homogenate in presence and absence of isoproterenol. In absence of isoproterenol, no significant change in TBARS level was observed after both phlorizin (Phz group) and ritonavir (RTV group) treatment in comparison to control. Significant (p < 0.05) increase in TBARS level was observed after addition of isoproterenol (ISO group). However, no significant change in TBARS level was observed in ISO group after phlorizin (ISO + Phz group) and ritonavir (ISO + RTV group) treatment in comparison to ISO group (Table 4).

**Table 4:** **TBARS levels in heart homogenate after treatment with different pharmacological agents in *in-vitro***

**Groups**	**TBARS (nmoles /mL homogenate)**
Control	8.61 ± 0.15
ISO	12.40 ± 0.15*
Phz	8.03 ± 0.12
RTV	8.23 ± 0.90
ISO + Phz	12.68 ± 0.58*
ISO + RTV	13.23 ± 0.50*

All the values were expressed Mean ± SEM, (N = 3) *p < 0.05 vs Control group.

## Discussion

Excessive release of catecholamine is associated with typical myocardial pathology under stressful conditions. Catecholamine and its metabolites play an important role in the pathogenesis of free radical-induced oxidative stress in heart. Although cardiotoxicity occurs primarily via adrenoceptor activation [14], increasing evidence suggests that it may also occur through oxidative mechanisms [15-17]. Dhalla et al. (1996) have reported that excess catecholamine affects calcium transport mechanism primarily via free radical mediated oxidative damage [17]. Thus β1-adrenoceptor antagonist and antioxidants may be indicated the best therapy for stress induced heart disease. Along with oxidative stress, increased cardiac glucose uptake was observed after catecholamine administration [18]. Glucose is also one of the major sources of energy in ischemic and stressed heart [3]. However, the effect of glucose transporter inhibition during catecholamine-induced cardiac injury was not observed previously. We administered phlorizin (SGLTs inhibitor) and ritonavir (GLUTs inhibitor) in isoproterenol-induced injured heart and observed their role in heart.

In the present study, we have observed a significant increase in heart weight and body weight ratio in ISO-induced mice. This increase in heart weight appears to be a hypertrophic response. Isoproterenol induced cardiac hypertrophy was also reported earlier [7]. The observed increase in the heart weight in ISO induced mice might be due to increase in protein synthesis and invasion of inflammatory cells in necrotic tissue [19]. Increased glucose uptake in heart along with increased oxidative stress in ISO administration might be responsible for cardiac hypertrophy. Association of cardiac hypertrophy and increased glucose uptake in heart was reported earlier [5,20]. Thus, both phlorizin and ritonavir (SGLT1 and GLUT inhibitor respectively) attenuated ISO induced cardiac hypertrophy might be via inhibiting glucose uptake in heart.

During myocardial injury cardiac membranes becomes leaky and results in higher serum level of creatine kinase (CK) and SGOT enzymes. These enzymes enter into the blood stream and thus increasing their concentration in the serum, and serve as the diagnostic markers of myocardial tissue damage [21-23]. Isoproterenol is well known cardiotoxic agent due to its ability to damage cell membrane. A significant elevated level of serum SGOT and CK after isoproterenol treatment was reported in mice earlier [7]. In the present study, we have observed elevated levels of serum SGOT and CK after ISO administration. Similar to ISO group, there were elevated activities of CK and SGOT in case of phlorizin. Unlike phlorizin, ritonavir showed complete cardioprotection by abrogating ISO effects.

Lipid peroxidation and endogenous antioxidants were also measured to confirm the myocardial oxidative stress after administration of isoproterenol. Isoproterenol produce quinones which react with oxygen to generate superoxide anions [O_2_ · -] and H_2_O_2_, which have damaging effects in cells [24]. ISO induced oxidative stress can be characterized by reduction of myocardial SOD, catalase and GSH along with a rise in myoacardial TBARS level [7,22,25]. It was previously reported that ISO could activates nitric oxide synthase (NOS) and increase the formation of reactive nitrogen species (RNS) during myocardial infarction [26]. Increased myocardial lipid peroxidation after isoproterenol appears to be the initial event of oxidative damage. In the present study, we have observed significant reduction of myocardial SOD and catalase activity, myocardial GSH and elevation of myocardial TBARS and nitric oxide, which confirms ISO-induced myocardial oxidative stress. Administration of phlorizin in ISO-treated mice only improved myocardial SOD, catalase and nitric oxide levels but no change in myocardial TABRS and GSH. Hence, phlorizin showed partial protection against ISO-induced myocardial oxidative stress. However, ritonavir normalized all oxidative stress parameters and thus showed complete protection against myocardial oxidative stress potentiated by isoproterenol. Although, the antioxidant effect of phlorizin in scavenging free radicals is reported earlier [27], no antioxidant property is reported so far by ritonavir.

To further confirm whether phlorizin and ritonavir have any antioxidant property, we did *in-vitro* antioxidant assay with heart homogenate. Our study showed no significant change in TBARS level, a marker of lipid peroxidation, after addition of phlorizin and ritonavir in heart homogenate in absence or presence of isoproterenol. This indicates that both phlorizin and ritonavir had no additional antioxidant effect in presence of isoproterenol. Thus the cardioprotective effect of phlorizin and ritonavir might be due to the inhibition of glucose uptake in heart.

ISO-induced myocardial necrosis is also confirmed by histopathological findings. ISO-treated heart showed the features of severe inflammation, the presence of inflammatory cells between the fibers, vacuolation of myocytes and few focal areas of myocytolysis. These all changes were previously reported in various ISO-induced myocardial necrosis models [23,25]. However, administration of either phlorizin or ritonavir showed no sign of cardiac necrosis in histopathology.

Catecholamine or isoproterenol generally increases heart rate and glucose uptake in heart. The reason of myocardial injury by isoproterenol might be through catecholamine induced free radical generation. It is possible that increased glucose uptake in heart might be responsible for more free radical generation and oxidative stress. Our present study showed that inhibition of cardiac glucose uptake through GLUT and SGLT1 blockers were beneficial for ISO-induced necrotic heart. We found partial protection by phlorizin, a SGLT1 inhibitor, in ISO-induced myocardial necrosis in mice. This protection might be possible through inhibition of cardiac glucose uptake through SGLT1 glucose transporter. Most important finding in our study is that ritonavir (GLUTs inhibitor) provides complete protection in ISO induced myocardial necrosis. It attenuated heart/body weight ratio, serum markers and oxidative stress. Hence ritonavir proved to be a better cardioprotective agent and prevents myocardial oxidative stress in ISO induced myocardial necrosis.

Thus our study concludes that non-specific GLUT inhibitor, ritonavir showed complete protection during ISO-induced myocardial necrosis. However, further studies should be carried out to find out whether ritonavir possess cardioprotection through additional effect unrelated to glucose transporter inhibition.

## Competing interests

The authors declare that there are no conflicts of interest.

## Authors’ contributions

PG, AK, YB, TNK, BS and MK carried out animal experimentation, biochemical estimation and statistical analysis of results. UKP did all histopathology work and its interpretation. SKB conceived the study, and participated in its design, coordination and drafted the manuscript. All authors read and approved the final manuscript.

## Authors’ information

PG, YB and BS are MS in Pharmacology (NIPER, Hyderabad), AK and TNK are Senior Research Fellow from Council of Scientific and Industrial Research, MK (PhD) is Senior Technical Assistant, UKP is a scientist in National Institute of Nutrition, Hyderabad and SKB (PhD) is Principle Investigator in the Division of Medicinal Chemistry and Pharmacology, Indian Institute of Chemical Technology (IICT), Hyderabad-500607, India.
